# Frequency and the risk factors of hepatitis C virus in pregnant women; A hospital based descriptive study in Gadap Town Karachi

**DOI:** 10.12669/pjms.335.12493

**Published:** 2017

**Authors:** Kausar Jilani, Bushra Zulfiqar, Qadir Bux Memon, Muhammad Faisal Fahim

**Affiliations:** 1 Dr. Kausar Jilani, MCPS, FCPS. Department of Gynecology & Obstetrics Unit-II, Al-Tibri Medical College & Hospital, Isra University, Karachi, Pakistan; 2 Dr. Bushra Zulfiqar, MCPS, FCPS. Department of Gynecology & Obstetrics Unit-II, Al-Tibri Medical College & Hospital, Isra University, Karachi, Pakistan; 3 Dr. Qadir Bux Memon, M.Phil. Department of Anatomy, Al-Tibri Medical College & Hospital, Isra University, Karachi, Pakistan; 4 Mr. Muhammad Faisal Fahim, M.Sc. Quality Enhancement Cell & Research (QEC&R), Al-Tibri Medical College & Hospital, Isra University, Karachi, Pakistan

**Keywords:** Pregnancy, HCV, Risk Factors

## Abstract

**Objective:**

To determine the frequency and the risk factors of hepatitis C virus in pregnant women at Al-Tibri Medical College & Hospital in Gadap Town Karachi.

**Methods:**

This was a descriptive cross sectional study conducted at Obstetrics & Gynecology OPD of Al-Tibri Medical College & Hospital, Isra University Karachi Campus from 10^th^ June to 10^th^ September 2016. A total of 400 pregnant women of 16-45 years of age, who came in outpatient department for antenatal checkup were selected for the study. The diagnosed cases of Hepatitis C were excluded from the study. Detailed history including age, parity, risk factor like history of transfusion, previous surgeries, vaginal deliveries was taken and relevant examination was performed. Patients on routine antenatal investigation if found to have Anti HCV positive on Immunochromatography Test (ICT) method were further confirmed by Elisa. A well designed proforma was used for data collection.

**Results:**

During the period of 3 months 400 women in antenatal clinic were tested for hepatitis C, out of which 27 (6.6%) were positive for HCV antibodies. The age of the women included ranges from 16-45 years. Thirteen (7.9%) pregnant women having HCV +ve antibodies fell in 26-30 years of age group. From 27 HCV +ve patients, 19 (70.3%) were multigravida & 8 (29.6%) were primigravida. Majority of the patients (77%) had history of injections.

**Conclusion:**

There is high prevalence of Hepatitis C infection among pregnant female in our setup. The possible risk factors are injection, blood transfusion and surgery.

## INTRODUCTION

Hepatitis C infection is one of the major health issue worldwide caused by hepatitis C virus. The virus can cause both acute and chronic hepatitis infection ranging in severity from a mild illness lasting for few weeks to serious life long illness such as cirrhosis, hepatocellular carcinoma and death. The mode of transmission is mainly parenteral and vertical. WHO estimates the prevalence of hepatitis c is 3% of the world population with more the 3 million new cases are being reported, representing a leading cause of liver cancer and transplant.^1^ In Pakistan, the prevalence of hepatitis C virus infection ranges from 8% - 15% in the general population with variations in different parts of the country.^2^ In Pakistan about 10 million people are infected with HCV within estimated population of 160,943,000.^3^ Being a vulnerable group, pregnant women are likely to be more infected.^4^

Prevalence of hepatitis C in pregnancy has been studied across Pakistan and is reported with the range of 3.27%-8.9%.^5^ HCV is transmitted readily by blood to blood contact as it is hepatotrophic virus.^6^ Viral hepatitis has increase risk of maternal complications during pregnancy and it is a notable reason of maternal mortality.^7^ In low resource countries, where there is lack of awareness and research orientation, the epidemiology and risk factors for HCV are poorly understood.^8^ The prevalence of HCV in population can be predicted by the risk factors associated with transmission of infection. These risk factors includes, blood products transfusion, occupational injury, surgery, injection and vertical transmission.^9^

The objective of this study was to find out the frequency of hepatitis C virus and its risk factors in pregnant women attending the Al- Tibri Medical College & Hospital.

## METHODS

This is a descriptive cross sectional, study and was conducted at Al-Tibri Medical College & Hospital (ATMC&H), Isra University Karachi Campus in Gynae & Obs (OPD) from 10^th^-June to 10^th^-September 2016. Approval of the study was taken from (IREC) Institutional Research Ethical Committee of ATMC&H. The sample size was calculated from the Software G Power version 3.0.10. The required sample size found to be 400 cases through one tailed t-test with alpha error of 0.05 and Power of test (1 – β err prob) was 0.8. Non-probability convenient sampling was used for the selection of patients. All pregnant women, age group 16-45 years irrespective of gestational age were included in this study. Non-pregnant patient and diagnosed cases of Hepatitis C were excluded from the study.

All pregnant women on their 1^st^ antenatal visit were selected for this study. Detailed history including age, parity, risk factor like history of transfusion, previous surgeries, vaginal deliveries etc., was taken from all respondents. Patients on routine antenatal investigation if found to have Anti HCV positive on ICT method were further confirmed by Elisa. A well designed proforma was used for data collection. An informed consent was taken from the patient. Data was analyzed through the SPSS version 20.0 software. The entire continuous variables (age and duration of pregnancy) were presented as mean ± SD. The entire categorical variables (Anti HCV ICT, Anti HCV Elisa) were shown in frequency and percentages.

## RESULTS

During the study period of 3 months, 400 pregnant women were tested for hepatitis C specific antibodies by Immunochromatographic technique (ICT). This test was positive in 39 patients, out of these 39 patients the diagnosis of hepatitis C was confirmed in 27 (6.6%) patients by Elisa method.

The age of the women included ranged from 16-45 years. Majority of the patient with HCV antibodies fell in 26-30 years of age group 13 patients, followed by 21-25 years 7 patients. Table-I. Out of 27 HCV positive patients 19 (70.3%) were multigravida and 8 (29.6%) were primigravida as shown in Table-II.

**Table-I:** Age distribution of study participants and Anti-HCV status.

*Age Group*	*Anti HCV Antibodies*		*Total*

	*Positive*	*Negative*	
16-20	3 (3.2%)	91 (96.8%)	94
21-25	7 (21.2%)	26 (78.8%)	33
26-30	13 (7.9%)	151 (92.1%)	164
31-35	3 (4.3%)	67 (95.7%)	70
>=36	1 (2.6%)	38 (97.4%)	39
Total	27 (6.8%)	373 (93.3%)	400

**Table-II:** Showing Anti Hepatitis C with Parity status.

*Parity*	*Anti HCV Antibodies*

	*Positive (n = 27)*
Multi	19 (70.3%)
Primi	8 (29.6%)
Total	27 (100%)

The distribution of risk factor among the patients of hepatitis C positive is shown in Table-III. Use of injection was seen as major risk factor which was present in 21 (77%) patients. History of blood transfusion was seen as second most common factor, 11 (40%), while history of surgery both major and minor was present in 6 (22%) of the patients.

**Table-III:** Risk factors for HCV transmission in hepatitis C patients (27).

*Risk factors for HCV transmission*	*Frequency*
Injection	21 (77%)
Blood Transfussion	11 (40%)
Surgery	6 (22%)
Injection + Surgery	3 (11.1%)
Injection + Blood Transfusion	10 (37%)
Surgery + Blood Transfusion	1 (3.7%)
Injection + Surgery + Blood Transfusion	1 (3.7%)

Many patients had more than one risk factor, 3 (11.1%) of the patients had history of both surgery and use of injection and 1 (3.5%) had history of surgery, blood transfusion and use of injection.

## DISCUSSION

Hepatitis C is endemic in developing countries like Pakistan. According to the Journal of Pakistan Medical Association (2003), the antibodies to hepatitis C virus were found to be 20 folds higher in developing countries than in developed countries.^10^ The overall incidence in Pakistan ranges between 4% - 25%.^11^

The anti HCV antibodies prevalence in pregnant patient was 6.6% in our study. The review of different studies conducted across Pakistan by Shah and Shabbir in 2002 reported the prevalence of HCV ranging from 0.7% - 20% in pregnant patient.^12^ One study conducted at Karachi by Shirazi and colleagues, showed the prevalence of hepatitis C as 9.2%,^13^ which is higher than this study. Kumar et al in 2007 reported prevalence of HCV among pregnant women was 1.03% which was remarkably less as compare to the results of different studies conducted in Pakistan.^14^ In an epidemiological study of 34,336 patients in Japan the prevalence of HCV was 7.1%^15^ which is comparable to this study while in one study of Turkey the prevalence of anti HCV was 2.4%^16^ which is significantly low as compared to our study.

In our study the highest sero-prevalence of anti HCV was in the age group of 26-30 years which is comparable to the study by Gul N et al in Ayub Medical College (25-35 years).^17^ Almost similar age group is reported by one Swiss study^18^ whereas Khattak ST (in Swat) reported the age group of 30-39 years with highest prevalence of hepatitis C.^19^ Duru MU et al in 2009 also showed the high prevalence of hepatitis C in 32-34 years.^20^ In current study majority of the patient having anti HCV antibodies were found to be multigravida and these findings are similar to the studies conducted by Awan et al and Ali et al.^21^,^22^

Regarding the risk factors for hepatitis C virus transmission in this study, 77% patients had history of taking Injectables while blood transfusion and surgeries were also found as common risk factor seen 40% and 22% respectively. Janjua et al in 2005 reported that injection overuse is very common in Pakistan and most parenteral medications are provided with previously used equipment. Data from other studies substantiate the finding of poor infection control and non-adherence to universal precaution at first level care facilities.^23^ Farhana et al showed that history of surgery was most significant risk factor for HCV infection.^24^ Another study conducted at Shifa International Hospital Islamabad found that past history of surgical procedure, blood transfusions are the most significant risk factors.^25^ A study from India in 2007 showed that majority of the study population 62% of women with HCV had no evidence of exposure to any common risk factor.^14^ A study from USA parenteral drug use was reported to be the major risk factor in majority of anti HCV antibodies positive cases.^26^

HCV screening in routine antenatal clinics are not universally considered but in developing countries due to lack of healthcare facilities as well as learning of healthcare staff, it should be carried out during antenatal checkups. This situation suggests that urgent efforts are needed to increase awareness about hepatitis C prevention and early treatment.

## CONCLUSION

In conclusion, there is high prevalence of Hepatitis C infection among pregnant female in our setup. The possible risk factors are injection, blood transfusion and surgery.

### Authors’ Contribution

**KJ:** Conceived the study, Manuscript Writing &final review of manuscript.

**BZ:** Did data collection, Design of study & Manuscript writing.

**MFF:** Did statistical analysis, editing & reviewing the manuscript.
